# The Many Faces of Grief: A Systematic Literature Review of Grief During the COVID-19 Pandemic

**DOI:** 10.1177/10541373211038084

**Published:** 2023-01

**Authors:** Ritesh M. Kumar

**Affiliations:** 30112 Indian Institute of Technology Roorkee, Roorkee, Uttarakhand, India

**Keywords:** COVID-19, grief, bereavement, mourning, systematic literature review, thematic analysis, qualitative synthesis

## Abstract

Coronavirus disease 2019 (COVID-19) pandemic has halted life all around the world. The disease, along with quarantine, social distancing, unemployment, and displacement, has led to myriad losses. There is a rising concern for the epidemic of grief that can result from these multiple losses. The present study aimed to investigate how grief is understood and discussed in the extant literature during the COVID pandemic. A systematic literature review was conducted using PsycArticles, Web of Science, and Scopus databases. The qualitative synthesis of 33 articles indicates that grief can be manifested at various levels such as grief for self, relational grief, collective grief, and ecological grief. Another theme emphasizes some of the factors that could intensify the grief process leading to prolonged grief disorder. A third theme relates to the focus of grief processes as experienced by individuals in different developmental periods from childhood to senescence. The study contributes theoretically by expanding our perception and understanding of varied forms of grief.

## Introduction

The novel coronavirus disease-2019 (COVID-19), first identified in Wuhan, of China, has spread around the world like a wildfire. On 11 March 2020, the World Health Organization (WHO) declared it a pandemic (WHO, 2020). The COVID-19 pandemic has halted life all around the world. Despite the approval and distribution of vaccines, the infection rate in many parts of the world is still high. Although some countries have begun to prepare for postpandemic life, others are still struggling to keep the death tolls low. The pandemic has resulted in the deaths of millions of people. It has impacted the lives of nearly everyone in some way or another. As of July 2021, total known cases of 191,657,167 and 4,111,838 deaths have occurred due to COVID (Worldometer, 2021). Besides a large number of deaths, it has also resulted in losses of multiple kinds. As the world quarantined itself, many people lost their employment and livelihood opportunities; at many places, hospitals turned their backs to non-COVID illnesses (Hamid & Jahangir, 2020); industries, schools, universities, and transport systems were shut down to contain the spreading infection. All of these resulted in losses and, consequently, grief responses that affected the physical and psychological health of the individuals.

Verdery et al. (2020) predicted that for every death due to COVID-19, nine people are expected to grieve in the United States. This estimated number will be higher for non-western collectivistic societies where close kinship ties and larger families are prevalent. A large number of individuals will find themselves mourning the loss of their loved ones. Petry et al. (2021) have suggested that these losses will result in “epidemic within epidemic.” What makes dying and grieving unique during the COVID pandemic is the voluntary and involuntary isolation from others, the inability to say goodbye or to organize a proper funeral for the deceased. COVID deaths are considered bad deaths because of circumstances surrounding the death, making the bereaving individuals prone to chronic and pathological grief (Carr et al., 2020). Moreover, for many bereaving individuals, their losses may not be duly recognized as the dead get reduced to mere statistics and numbers displayed on screens. These conditions prevent mourning and could complicate the grieving process. Such disruptions in the grieving process could lead to prolonged grief disorder (PGD), a newly added psychological disorder, by the International Classification of Diseases (ICD).

Several studies have confirmed that the COVID pandemic, with its multiple losses in different spheres of life, has adversely affected the mental health of individuals (Kumar & Nayar, 2020; Vindegaard & Benros, 2020). Shigemura et al. (2020) have emphasized the effects of COVID on fear, anxiety, loneliness, emotional outburst, and sleep disturbances. Issues with anxiety, sleep, stress, and suicide due to COVID is also discussed by Sher (2020). The fear associated with infection and the anticipation of its severity and death are possible causes for these mental traumas. Pandemic effects can be more severe in the form of panic attacks, depression, paranoia, and suicide (Ornell et al., 2020). This widespread negative impact of COVID on mental health makes it imperative to explore its influence, particularly on bereaving individuals, and make amends by suggesting psycho-social interventions.

The mental health of bereaving individuals is likely to be severely affected during the pandemic. This life-threatening disease has eclipsed appropriate intervention strategies for the bereaving individuals. Bereavement is associated with several physical and psychological health consequences (Stroebe et al., 2007). Although the terms grief and bereavement are often used interchangeably, there are some differences between them. “Bereavement is the experience of losing a loved one, and grief is the natural response to this loss” (Goveas & Shear, 2020, p. 1120).

Several theories of grief have emerged that provide different perspectives for understanding bereavement. Kubler-Ross and Kessler (2005) divided grief into five stages—denial, anger, bargaining, depression, and acceptance. Scholars have criticized such categorization (Maciejewski et al., 2007) as it tends to disregard cultural differences in grieving and conceptualizes grieving in stages that may not be true for everyone. Individuals do not necessarily progress through these stages; they may randomly jump from one stage to another. The phase model is another model for understanding grief. Based on attachment theory, it describes the process of grieving as consisting of four phases, “phase of numbing,” “phase of yearning and searching,” “phase of disorganization and despair,” and “phase of reorganization” (Bowlby, 1980, pp. 85–96). A third model for understanding grief is the dual-process model developed by Stroebe and Schut (2010), described as a model of coping with bereavement. According to this model, grieving consists of two categories of stressors, loss versus restoration-oriented stressors. While loss-oriented stressor is concerned with the bereaved person's focus on the experience of the loss, the restoration-oriented stressors are concerned with the focus on the necessary stressors to reorient the world without the deceased person. These theories conceptualize grief in relational terms, i.e., grief for the loss of a loved one. However, as mentioned earlier, the pandemic and massive loss of life are unique in some aspects. This uniqueness is likely to influence grief responses.

A more relevant model for understanding grief during the COVID pandemic is Chater’s (2020) “*Your world and the ball of grief*.” This model claims that one may not completely get over grief and its painful effects. However, as one navigates through their world, their experiences expand, and the grief begins to appear smaller and more manageable. The model also claims that a person can experience multiple griefs simultaneously. This aspect makes it particularly relevant for the pandemic situation as individuals experience multiple losses such as loss of loved ones, employment and livelihood, identity, and significant life events.

In the past few months, several studies have emerged on grief during the pandemic. These studies provide us with valuable insights into the process of grief and bereavement at such a distressing time. However, these studies remain dispersed. There is a need to synthesize these studies to understand the grieving process. The current study was carried out to synthesize the extant literature on grief and bereavement during the COVID pandemic. The research question posed in this regard was: How does the extant literature distinguish the grief and bereavement of COVID-19 from other periods and situations? What are the underlying themes in the grief and bereavement literature of the COVID pandemic period?

## Method

A systematic literature review was employed to answer the research question. A PRISMA model was used for the selection of studies. PsycArticles, Web of Science, and Scopus databases were searched for relevant studies using *COVID-19 and Grief* and *COVID-19 and Bereavement* as the search terms. Altogether 201 records were extracted (as of April 2021). The abstract of each of these records was assessed for relevance. Duplicate records and records that were not linked to grief during the COVID pandemic were removed. This assessment resulted in identifying 33 records that were relevant in answering the questions of the present study. A qualitative synthesis of these records was carried out to answer the research question.

## Results

We employed thematic analysis to identify the major themes discussed by authors in the extant literature. THE following major themes were identified:

### Many Faces of Grief

Authors have written about different forms of grief that can be organized at different levels. The four levels of grief discussed in the literature are—bereavement for self, grief for the loss of a loved one (relational grief), collective grief, and ecological grief.

#### Grief for Self

Albuquerque et al. (2021) write about bereavement for self, which could result from the loss of life events, employment, milestones, and financial security due to the direct or indirect effects of the pandemic. These authors particularly emphasize the disenfranchisement of bereavement of self for losses that may not be socially acknowledged. Nair and Banerjee (2020) write in this regard that “[t]raditionally associated with ‘loss’ varied connotations of grief might arise … It can classically range from losing a loved one to the perceived loss of autonomy and dignity, especially in the marginalized populations” (p. 1). Grief for self can also result from an anticipated death due to a decline in one's health conditions (Ishikawa, 2020). Masiero et al. (2020) write about the grief of self in terms of loss of identities that could result from loss of one's career and employment opportunities.

#### Relational Grief

Relational grief here refers to grief in its traditional usage of the term, i.e., grief for losing a loved one. Different forms of grief are discussed at this level, such as complicated grief, ambiguous grief, anticipatory grief, and disenfranchised grief. Although these forms of grief could be present at other levels, they are especially emphasized for bereavement for losing a loved one.

Disenfranchised grief is defined in the literature as losses that go unacknowledged by society and community. Wallace et al. (2020) write that reducing human deaths to mere numbers and statistics, failure to socially acknowledge the deceased, and restrictions surrounding funeral arrangements during the COVID pandemic could lead to disenfranchised grief for the bereaving individuals.

Complicated grief results from multiple stressors and is characterized by excessive rumination, alienation, hopelessness, and intrusive thought for the dead (Wallace et al., 2020). These authors write that factors associated with the COVID pandemic, such as social distancing, lack of support, and inability to prepare for death, can result in complicated grief. Scheinfeld et al. (2021), in their survey study, found that the multiple losses that people are experiencing lead to the compounding of grief.

Walsh (2020) has written about the concept of ambiguous loss. The author writes that “ambiguities persist about how the virus is spread and whether a death was due to coronavirus. Lack of clarity about the diagnosis, symptoms, and severity can impede getting emergency care” (p. 5). Scheinfeld et al. (2021) have also written about ambiguous loss:

…ambiguous loss may be experienced in a variety of loss types beyond death, and that it can include intangible forms of loss as well, such as losing time with family and friends, events, milestones, and routine activities or “normalcy” in everyday life (p. 7).

Anticipatory grief is another form of grief defined as a bereavement for an anticipated loss. Singer et al. (2020) term such a loss as preloss grief (PLG) and define it as a grief response that occurs as someone struggles to come to terms with both the potential loss of a loved one and the changes that result from a loved one’s health decline. Similarly, due to governmental policies and restrictions (lockdowns and quarantines), the process of grief and bereavement could be delayed leading to suspended or delayed grief (Albuquerque & Santos, 2021).

#### Collective Grief

A large number of deaths in the community results in grief beyond individual bereavement and one that is shared by the larger community. Chater (2020) has talked about grief at a more communal level, collective grief, for bereavement for the loss of many lives in one's community, nation, and worldwide. Masiero et al. (2020), in this respect, talk about collective trauma in the context of COVID-19.

#### Ecological Grief

Crossley (2020) defines ecological grief as “grief felt in relation to experienced or anticipated ecological losses, including the loss of species, ecosystems, and meaningful landscapes due to acute or chronic environmental change” (p. 540). According to the author, such bereavement expands the concept of the grief process from human to larger ecology. Unfortunately, this extension is often not acknowledged in society leading to the disenfranchisement of grief. The author further writes that:

[i]t is my prediction that the immediacy of the COVID-19 pandemic will, for many people, bring hitherto unresolved feelings relating to ecological grief out into the open. The escalation of climate consciousness, agitation and anxiety that we saw last year has, in a way, reached a bewildering and unexpected climax in the form of this pandemic. (Crossley, 2020, p. 540)

### Factors Influencing the Severity of Grief

Several factors have been identified in the literature that could influence grief severity during the pandemic. These can be primary stressors, secondary stressors, health complications, relationship ambivalence, and the delegitimization of the loss. Fernandez and Gonzalez-Gonzalez (2020), Tang and Xiang (2021), and Walsh (2020) have written about the effects of primary stressors on the severity of grief responses. Primary stressors are the ones that directly result from the COVID, such as the loss of life or livelihood and multiple losses. Such primary stressors have been identified as an essential precursor of PGD. Besides the primary stressors, the pandemic is also responsible for other stressors such as social distancing, quarantine, prevention of gatherings, funeral practices, travel, and recreation. Kokou-Kpolou et al. (2020) write that multiple deaths and losses, restrictions on meeting the dying, and organizing funeral practices are likely to result in pathological grief, leading to an increase in PGD.

Eisma et al. (2021), Nair and Banerjee (2020), and Wallace et al. (2020) write that these secondary stressors add to the primary stressors and complicate the grieving process. Tang and Xiang (2021) write about health complications such as coronary heart disease, diabetes, and other illnesses that can increase the vulnerability of individuals and make the grief responses severe. However, Scheinfeld et al. (2021) write about the role of delegitimization of grief in intensifying the grief responses.

### Development Period and the Experience of Grief

Some authors have focused on grief and bereavement in different age groups, particularly children and adolescents, during the COVID pandemic.

#### Children

Albuquerque and Santos (2021) write that children could lack a proper support system during the grieving process during this pandemic. This could be because of the loss of loved ones to COVID or because their support systems are themselves in the grieving process. Grief could be manifested through somatic symptoms and maladaptive behaviors. Bereavement at such distressing times could also result in delayed grief. Santos et al. (2021) have made a further distinction in the grief experience of preschool children and school-age children. According to these authors, an important point that distinguishes the experience of grief among preschool children and children is the knowledge of the irreversibility of death. The preschool children do not understand that death is irreversible, which can result in guilt and regret. However, older children do understand that death is irreversible.

#### Adolescence

Santos et al. (2021) write that adolescents understand death as “irreversible, universal, and nonfunctional” (p. 2). The grieving process of adolescence resembles that of adults. Weinstock et al. (2021) write that grief during the pandemic could disrupt identity formation among adolescents. Further, they may experience problems at the workplace, difficulties in educational achievements and aspirations, and an increased risk of psychological disorders. Weinstock et al. (2021) further suggest that adolescents may be vulnerable to develop complicated grief due to the lack of an adequate support system.

#### Adults

The disproportionate influence of the pandemic on the disadvantaged community is discussed by many scholars. Nair and Banerjee (2020) write that the socio-cultural contexts shape the experience of grief during the pandemic. Authors such as Moore et al. (2020) have raised concerns regarding the racial and ethnic minorities in different parts of the world. It is predicted that these marginalized and disadvantaged communities are more vulnerable to complicated and PGDs.

#### Elderly

Authors have emphasized the anticipatory grief for the elderly population. For instance, Nair and Banerjee (2020) write that “elderly (geriatric population), especially when living alone or terminally ill, already being frail and vulnerable, are undergoing anticipatory grief” (p. 1). A similar discussion for anticipatory grief is also expressed in Ishikawa (2020). However, the elderly population may lack an adequate support system for grieving during the pandemic leading to complicated grief. These authors write:

Many older adults are already grieving the loss of independence, social connectedness, financial security, and access to necessities and supports. As they mourn these losses, they fear the even more overwhelming loss of health and life (their own or loved ones’), the ability to plan for the future, and control, whether real or perceived, over their own lives. (Ishikawa, 2020, p. S85)

Goveas and Shear (2020) write that certain risk factors such as sudden, unexpected, and preventable deaths, visiting restrictions, and financial insecurities make older adults vulnerable to develop PGD.

### The Analogy Between War and Pandemic

Sowden et al. (2021) have explored how the media has compared the COVID situation as “war with an invisible enemy.” The authors write that using patriotic language was invoked in media reports to create personal responsibility and propel for action by indulging in COVID-appropriate behaviors. An important similarity between pandemic and war is the massive loss of lives. The mourning and grief resulting from these deaths and losses are compared with losing lives in war situations. Such an analogy between war and pandemic is not limited to media and rhetoric speeches. Hamid and Jahangir (2020), in their research article, made a similar analogy. Furthermore, Fernandez and Gonzalez-Gonzalez (2020) write that “an analysis of the information gathered makes it plain that the sensations of grief, emptiness, sadness, and anxiety are compared to wartime.” Maddrell (2020) writes in this regard that (Table 1):

**Table 1.** Summary of Included Studies.

Study	Sample/population	Nature of article	Relevant findings/discussions
Eisma et al. (2021)	People bereaved through COVID-19, natural causes and unnatural causes	Original article	Multiple stressors result in severe grief; social distancing hampers grief reactions; severe grief reactions for COVID-related bereavement; expectedness explained the difference between grief due to natural cause and COVID-related death
Sowden et al. (2021)	Media reportage of COVID-related death	Original article (qualitative synthesis)	The war analogy provoked by three themes: uncontrollable, unknown, enemy; call for actions; and mourning and loss, compounded by multiple losses and multiple deaths in the family
Albuquerque et al. (2021)		Opinion	Grief that is unrecognized, socially invalidated, and unprocessed causes disenfranchisement. It can be external disenfranchisement or self-disenfranchisement of grief.
Tang and Xiang (2021)	422 Chinese participants	Original article, cross-sectional survey	PGD was higher for COVID-related deaths. The personal traumatic level of loss was more significant for grieving than the unexpectedness of death. The relationship quality determines grief severity.
Albuquerque and Santos (2021)	Children and adolescents experiencing grief	Opinion	Children are equally affected by grief for their loss during the pandemic. Death-related cues could hamper the restoration coping of children, and support systems may be absent at pandemic times.
Weinstock et al. (2021)	Adolescents experiencing grief	Perspective	“Grief could disrupt identity quest among adolescents.” Lack of a support system could complicate grieving. Grief can result in physical, behavioral, and socio-cognitive issues.
Stroebe and Schut (2021)		Systematic literature review, qualitative synthesis	A rise in PGD due to increased mental difficulties or disorders in the grieving process is predicted.
Petry et al. (2021)		Opinion	Grief is suggested to result in an epidemic. Different forms of grief—complicated grief, prolonged grief, and disenfranchised grief could be manifested.
Santos et al. (2021)	Grief responses among children and adolescents	Case report	The grieving process is different for children and adolescents. Preschool children were unable to understand death as irreversible. Adolescents can understand death as irreversible, universal, and nonfunctional.
Breen et al. (2021)	Adults from the United States bereaving due to COVID pandemic	Original article	Pandemic grief could result in functional impairment for bereaved individuals—a requirement of cost-effective strategies to deal with disordered grief.
Nair and Banerjee (2020)		Letter to the editor	Grief could emerge from the loss of a loved one and the loss of autonomy, dignity, life events, and livelihoods. In addition, isolation and loneliness are major factors amplifying grief; thus, social integration and connectedness are important tools to deal with grief.
Pirnia et al. (2020)		Letter to the editor	Grief can result in a “ripple effect,” which can influence other family and community members.
Eisma et al. (2020)		Letter to the editor	An increase in PGD rates is predicted due to the circumstances of many COVID-19 deaths. The author reason that PGD symptoms increase when “deaths are unexpected, traditional grief rituals are absent and physical, social support is lacking.”
Ishikawa (2020)	Older adults	Commentary	Older adults are expected to experience anticipatory grief due to lost independence, lost social connectedness, lack of financial security, and lack of access to necessities and supports.
Maddrell (2020)		Commentary	Multiple losses emerge during pandemic personal, economic, social, and political. Abandoning of dying and the dead and the quantification of death indicates dispensability. Certain social groups are more vulnerable than others. National rhetoric silences personal and collective grief and anger.
Kokou-Kpolou et al. (2020)		Commentary	Multiple deaths could lead to “bereavement overload,” a distressing overflow of grief that hampers coping capacity. Moreover, excessive and collective accumulation of deaths could deny recognition of individual grief—rise in traumatic, disenfranchised, and chronic grief during the pandemic.
Carr et al. (2020)		Commentary	COVID deaths are bad deaths due to circumstances surrounding them. Although bad deaths are distressing in themselves, multiple stressors during a pandemic could amplify this distress.
Crossley (2020)		Commentary	Presence of deep anxiety about the worsening environmental and climatic conditions. Ecological grief is the “grief felt in relation to experienced or anticipated ecological losses, including the loss of species, ecosystems and meaningful landscapes due to acute or chronic environmental change.”
Singer et al. (2020)		Commentary	PLG develops in response to the anticipation of the loss of a loved one. It includes confusion about one's role in life, emotional distress, and feeling shocked.
Goveas and Shear (2020)	Older adults	Case history	Certain factors hamper grief process, such as personal characteristics of bereaved person, relationship with deceased, circumstances surrounding death. Sudden, unexpected, preventable death, visiting restrictions, insecurities, and financial worries could intensify PGD.
Wallace et al. (2020)		Original article	Anticipatory grief is mourning for expected death. Physical, mental, and social consequences of isolation may result in complicated grief. Treating death as a mere statistic can cause disenfranchised grief.
Eisma and Tamminga (2020)	Cross-sectional sample of 1600 bereaved individuals	Empirical article	Grief severity was not significantly different during and immediately before the pandemic. However, experiencing a recent loss during the pandemic increased grief severity than a loss before the pandemic.
Vázquez Bandín (2020)		Original article	It is the living who are left alone, frustrated, and powerless with an unfinished history and task of seeing the dying, saying goodbye, organizing their funeral, and grieving for the dead.
Walsh (2020)		Original article	COVID deaths could be distressful for the whole family. Multiple losses—loss of physical contact, livelihood, financial security, hopes, and normalcy are experienced. Families are distressed by emotional, relational, and functional impact of multiple stresses.
Burrell and Selman (2020)		Systematic literature review	Inconclusive evidence for the effects of funeral participation on mental health. Findings suggest that restrictions to funeral practices do not necessarily negatively hamper the bereaved: depth, meaning, and sense of control determine helpfulness of the funeral.
Fernandez and Gonzalez-Gonzalez (2020)	Critical discourse analysis of mass media information	Original article	Individuals who recovered in ICUs share some commonalities – cognitive, affective, and spiritual. Gender differences in the grieving process, while women seek support and are expressive; men tend to rationalize. Grieving process during pandemic is often compared to wartime.
Hanna et al. (2021)	19 bereaving individuals from the United Kingdom	Original article; qualitative study	Staying connected with the family members, availability of support networks, clear communication, and support from health professionals are helpful in bereavement.
Scheinfeld et al. (2021)	257 participants from North America	Original article	Ambiguous loss can include “loss of time with family and friends, events, milestones, routines, and the normalcy of everyday life.” The findings suggest that individuals can feel “guilt and de-legitimization” for their losses that hamper support-seeking behaviors.
Hamid and Jahangir (2020)	17 interviews with individuals bereaving during pandemic	Original article; qualitative study	Unavailability of proper medical care for non-COVID individuals is responsible for many deaths. Stigma surrounding the diseases has caused much distress. Dying alone was traumatic for the survivors.
Menichetti Delor et al. (2021)	Reports, interviews, and peer group discussions with psychologists	Qualitative study	Themes identified for experiences of family members: absence of death rituals, solitary, unexpected and fast, unfair, unsafe, and co-existent with other stressors. Themes identified for families’ needs: to give meaning, to express emotions, to say goodbye, to remember, and to solve practical issues.
Chater (2020)		Blog post	Grief responses could emerge in the absence of personal loss due to collective grief. Re-grief is experienced when past bereavements re-emerge due to specific triggers such as mass losses during the pandemic.
Masiero et al. (2020)		Commentary	COVID-19 can be viewed as individual and collective trauma. Loss of self/identity, self-efficacy, self-esteem could result from job loss, economic uncertainty, caring strains.
Moore et al. (2020)	Case report of four African American individuals	Case report	Discrimination faced by Black communities during COVID: health professionals denied their complaints about symptoms. Black communities from all generation periods have experienced discrimination.

Note: COVID-19 = coronavirus disease 2019; PGD = prolonged grief disorder; PLG = pre-loss grief.

In the UK, governmental discourses of being at “war” against the virus have demanded national unity, gagging criticism of the government; likewise, the trope of heroic health and key workers has prefigured their deaths as unavoidable and sacrificial. This national rhetoric serves to silence and disenfranchise personal and collective grief—and anger—in response to the individual, key worker, racialized minority, and total deaths. (Maddrell, 2020, p. 109)

## Discussion

It is evident from the identified themes that experts have talked about different levels and forms of grief during the pandemic. From a highly personal grieving for one's self (bereavement for self) (Albuquerque et al., 2021) due to the effects of being quarantined, lockdown, loss of work, social isolation, loss of life events and milestones, to a higher order of grief manifested for the loss of the natural environment (ecological grief) (Crossley, 2020). Two other levels of grief are described: one is the relational grief, that is, grief for the loss of a loved one, and the other is collective grief, that is, grief with a more shared and communal orientation for the massive deaths caused in communities and countries. Within each of these four levels, grief could be manifested in different forms, such as acute grief, prolonged grief, complicated grief, disenfranchised grief, anticipatory grief, ambiguous grief, and delayed grief. Although Eisma et al. (2021) did not find any significant difference in grief for those in bereavement during the pandemic and just before the pandemic, their findings indicated that individuals who were bereaved during the pandemic experienced severe grief reactions than those individuals who were bereaved before the pandemic. They conclude that this severity could lead to disordered forms of grief, such as PGD.

Similarly, although some authors have emphasized that restrictions on funeral practices could influence mourning and grief, leading to complicated grief, Burrell and Selman (2020) did not find any conclusive evidence for the effect of funeral participation in their systematic literature review on mental health and bereavement outcomes. However, the studies included in Burrell and Selman (2020) were from the nonpandemic period when the engagement into funeral practices was determined by choice. An essential difference in funeral practices during the pandemic is the external restrictions and limitations imposed by the state and authorities.

Although some authors have drawn inconclusive evidence for the role of funeral restrictions or significant differences in the severity of grief during pandemic times, the majority of studies have emphasized that the multiple losses that people have experienced, and the nature and context of death along with restrictions on meeting and funeral is likely to increase PGD. These losses are expected to compound the bereavement process. Although Eisma et al. (2021) recognized the expectedness of death as an essential distinguishing factor for grief resulting from COVID death and death due to natural causes, Tang and Xiang (2021) have argued that “the subjective traumatic level of stress” is more significant than the unexpectedness of death for the severity of grief responses.There is a concern among mental health professionals that nonrecognition of the dead, their reduction to mere statistics, and the ambiguity surrounding the death are likely to result in disenfranchised and delayed grief responses. Moreover, for individuals in different life stages, from preschool children to the elderly, the process of grieving may vary. Among children, somatic responses, anger, and behavioral issues are expected to result from the grieving process. Also, marginalized communities based on race, class, region, and religion are likely to bear a greater burden for the grief resulting from this pandemic. Overall, there is consensus in the literature that losses during the pandemic will result in an epidemic of grief. The response of mental health professionals will play a significant role in the grief epidemic.

### Implications

The study has theoretical implications as it expands our perception and understanding of grief. When we think of grief, we usually perceive it in linear relational terms, that is, losing a loved one. The findings of the present study argue that grief can have multiple levels and forms. The findings expand the grief model “your world and the ball of grief” (Chater, 2020) by suggesting that the various balls of grief can be organized at varying levels and intensities with the potential to overlap and become severe. Another implication of the study is that it attempts to organize the extant literature trying to thread through the common themes in studies on grief during the pandemic.

The study also has some applied clinical implications. In the therapy settings, relational grief and associated complications such as PGD are likely to be recognized and intervened. However, other grief forms such as grief for self, collective grief, and ecological grief may be ignored. The present study argues that these forms of grief are equally important and require interventions similar to relational grief. Further, for each of these levels of grief, anticipatory, acute, compounded, complicated, delayed, and disenfranchised grief could be present, making the person vulnerable to grief disorders such as PGD. Collective grief requires collective community-based actions. Involving the personals and professionals from different spheres such as medical and media staff, police, community leaders, and volunteers by training them to provide some basic early interventions can prevent grief from becoming complicated and severe. It is also recommended that active community programs through online modes be encouraged to deal with the mental health impact of both COVID and the associated grief.

Another applied implication of the study is for the grief interventions for individuals in different developmental periods. Bereaving individuals in different developmental periods require different interventions. The mode of therapy will have to be adjusted accordingly. For instance, for the elderly, it is essential to explore anticipatory grief responses, whereas, for young children, intervention for guilt associated with grief will be beneficial. Overall, the study emphasizes the importance of grief interventions and recommends joint actions of individuals from different spheres of society.

### Limitations

Although the study provides us with insight into the major themes discussed in the literature on grief during the pandemic, there are some limitations to the present study. Firstly, some of the articles included in the study were not original research articles but commentaries, opinions, and letters to the editor. Despite this, these opinions do matter as they come from experts working in grief and bereavement. It is assumed that these experts comprehend the grief process during the current pandemic situation deeply, and given the emergency of the context, their expertise is much required. Secondly, the selection of articles was made from the Web of Science, PsychArticles, and Scopus databases. Although these databases contain a large number of materials, these are not an exhaustive list. Likely, other important manuscripts which suffered publication bias did not get included in the present study.

Future studies can explore the applied aspects of grief interventions for bereaving individuals. Due to the unique situation of the pandemic, the social distancing, and isolation, the way we conduct therapy is significantly affected. For most professionals, the face-to-face therapy is replaced by teletherapy. Some relevant questions that can be explored in future studies are—how have these changes influenced grief interventions? What are the recommendations for grief intervention at such distressing times and are they effective? Answers to these questions will be beneficial for effective grief interventions in the COVID context.

## Conclusion

The COVID pandemic has placed us in a strange and distressing context. As mentioned initially, a total number of 191,657,167 COVID positive cases and 4,111,838 deaths due to COVID have been recorded as of July 2021 (Worldometer, 2021). Although the disease itself has consumed millions of lives worldwide, factors associated directly or indirectly with it have caused other kinds of devastations to the lives of individuals. Through qualitative synthesis of the extant literature on grief during the pandemic, the present study identified the many different faces of grief that are evident during the pandemic. This finding challenges the conceptualization of grief in mere relational terms. The study also identified certain factors responsible for causing severe grief responses. Additionally, the review suggests that grief responses may vary depending on developmental period and the socio-cultural and economic contexts in which the individual is embedded. Moreover, the grief responses as expressed in different developmental age groups are also explored.

## References

[bibr1-10541373211038084] Albuquerque S. Santos A. R. (2021). In the same storm, but not on the same boat”: Children grief during the COVID-19 pandemic. Frontiers in Psychiatry, 12, 638866. 10.3389/fpsyt.2021.638866 PMC7870701 33574777

[bibr2-10541373211038084] Albuquerque S. Teixeira A. M. Rocha J. C. (2021). COVID-19 and disenfranchised grief. Frontiers in Psychiatry, 12, (February), 10–13. 10.3389/fpsyt.2021.638874 PMC7907151 33643101

[bibr3-10541373211038084] Bowlby J. (1980). Attachment and loss; Vol 3: Loss. Basic Books.

[bibr4-10541373211038084] Breen L. J. Lee S. A. Neimeyer R. A. (2021). Psychological risk factors of functional impairment after COVID-19 deaths. Journal of Pain and Symptom Management, 61(4), E1–E4. 10.1016/j.jpainsymman.2021.01.006 33476753

[bibr5-10541373211038084] Burrell A. Selman L. E. (2020). How do funeral practices impact bereaved relatives’ mental health, grief and bereavement? A mixed methods review with implications for COVID-19. Omega-Journal of Death and Dying, 1–39. 10.1177/0030222820941296 PMC9185109 32640878

[bibr6-10541373211038084] Carr D. Boerner K. Moorman S. Carr D. (2020). Bereavement in the time of coronavirus: Unprecedented challenges demand novel interventions. Journal of Aging & Social Policy, 32(4-5), 425–431. 10.1080/08959420.2020.1764320 32419667

[bibr7-10541373211038084] Chater A. (2020). Let's talk about death openly. The Psychologist, 33, 23–25. Retrieved from: https://thepsychologist.bps.org.uk/when-world-grieving-please-dont-walk-eggshells

[bibr8-10541373211038084] Crossley E. (2020). Ecological grief generates desire for environmental healing in tourism after COVID-19. Tourism Geographies, 22(3), 536–546. 10.1080/14616688.2020.1759133

[bibr9-10541373211038084] Eisma M. C. Boelen P. A. Lenferink L. I. M. (2020). Prolonged grief disorder following the coronavirus (COVID-19) pandemic. Psychiatry Research, 288, 113031. 10.1016/j.psychres.2020.113031 PMC7194880 32360895

[bibr10-10541373211038084] Eisma M. C. Tamminga A. (2020). Grief before and during the COVID-19 pandemic: Multiple group comparisons. Journal of Pain and Symptom Management, 60(6), E1–E4. 10.1016/j.jpainsymman.2020.10.004 PMC7553097 33065207

[bibr11-10541373211038084] Eisma M. C. Tamminga A. Smid G. E. Boelen P. A. (2021). Acute grief after deaths due to COVID-19, natural causes and unnatural causes: An empirical comparison. Journal of Affective Disorders, 278, 54–56. 10.1016/j.jad.2020.09.049 32950843 PMC7487144

[bibr12-10541373211038084] Fernandez O. Gonzalez-Gonzalez M. (2020). The dead with no wake, grieving with no closure: Illness and death in the days of coronavirus in Spain. Journal of Religion & Health, 1–19. Published online August 20, 2020. 10.1007/s10943-020-01078-5 PMC7439634 32816226

[bibr13-10541373211038084] Goveas J. S. Shear M. K. (2020). Grief and the COVID-19 pandemic in older adults. American Journal of Geriatric Psychiatry, 28(10), 1119–1125. 10.1016/j.jagp.2020.06.021 PMC7320675 32709542

[bibr14-10541373211038084] Hamid W. Jahangir M. S. (2020). Dying, death and mourning amid COVID-19 pandemic in Kashmir: A qualitative study. Omega-Journal of Death and Dying, 1–26. 10.1177/0030222820953708 32862778

[bibr15-10541373211038084] Hanna J. R. Rapa E. Dalton L. J. Hughes R. McGlinchey T., Bennett K. M. Donnellan W. J. Mason S. R. Mayland C. R. (2021). A qualitative study of bereaved relatives’ end of life experiences during the COVID-19 pandemic. Palliative Medicine, 35(5), 843–851. 10.1177/02692163211004210 33784908 PMC8114449

[bibr16-10541373211038084] Ishikawa R. Z. (2020). I may never see the ocean again: Loss and grief among older adults during the COVID-19 pandemic. Psychological Trauma: Theory, Research, Practice, and Policy, 12(S1), S85–S86. 10.1037/tra0000695 32551777

[bibr17-10541373211038084] Kokou-Kpolou C. K. Fernández-Alcántara M. Cénat J. M. (2020). Prolonged grief related to COVID-19 deaths: Do we have to fear a steep rise in traumatic and disenfranchised griefs? Psychological Trauma: Theory, Research, Practice, and Policy, 12(S1), S94–S95. 10.1037/tra0000798 32525367

[bibr18-10541373211038084] Kubler-Ross E. Kessler D. (2005). On grief and grieving: Finding the meaning of grief through five stages of loss. Simon and Schuster.

[bibr19-10541373211038084] Kumar A. Nayar K. R. (2020). COVID-19 and its mental health consequences. Journal of Mental Health, *30*(1), 1–2. 10.1080/09638237.2020.1757052 32339041

[bibr20-10541373211038084] Maciejewski P. K. Block S. D. Prigerson H. G. (2007). An empirical examination of the stage theory of grief. JAMA-Journal of the American Medical Association, 297(7), 716–723. 10.1001/jama.297.7.716 17312291

[bibr21-10541373211038084] Maddrell A. (2020). Bereavement, grief, and consolation: Emotional-affective geographies of loss during COVID-19. Dialogues in Human Geography, 10(2), 107–111. 10.1177/2043820620934947

[bibr22-10541373211038084] Masiero M. Mazzocco K. Harnois C. Cropley M. Pravettoni G. (2020). From individual to social trauma: Sources of everyday trauma in Italy, the US and UK during the COVID-19 pandemic. Journal of Trauma & Dissociation, *21*(5), 513–519. 10.1080/15299732.2020.1787296 32654633

[bibr23-10541373211038084] Menichetti Delor J. P. Borghi L. Cao di San Marco E. Fossati I. Vegni E. (2021). Phone follow up to families of COVID-19 patients who died at the hospital: Families’ grief reactions and clinical psychologists’ roles. International Journal of Psychology, 10.1002/ijop.12742 PMC8013378 33511652

[bibr24-10541373211038084] Moore S. E. Jones-Eversley S. D. Tolliver W. F. Wilson B. L. Jones C. A. (2020). Six feet apart or six feet under: The impact of COVID-19 on the Black community. Death Studies, 1–11. 10.1080/07481187.2020.1785053 32609079

[bibr25-10541373211038084] Nair V. S. Banerjee D. (2020). The heterogeneity of grief in India during coronavirus disease 2019 (COVID-19) and the national lockdown. Asian Journal of Psychiatry, 54, 102249. 10.1016/j.ajp.2020.102249 PMC7326433 32659656

[bibr26-10541373211038084] Ornell F. Schuch J. B. Sordi A. O. Henrique F. Kessler P. (2020). “Pandemic fear” and COVID-19: Mental health burden and strategies. Brazilian Journal of Psychiatry, 42(3), 232–235. 10.1590/1516-4446-2020-0008 32267343 PMC7236170

[bibr27-10541373211038084] Petry S. E. Hughes D. Galanos A. (2021). Grief: The epidemic within an epidemic. American Journal of Hospice & Palliative Medicine, 38(4), 419–422. 10.1177/1049909120978796 33280398 PMC7723733

[bibr28-10541373211038084] Pirnia B. Dezhakam H. Pirnia K. Malekanmehr P. Rezaeian M. (2020). Grief of COVID-19 is a mental contagion, first family suicide in Iran. Asian Journal of Psychiatry, 54, 102340. 10.1016/j.ajp.2020.102340 PMC7834061 32777756

[bibr29-10541373211038084] Santos S. Sa T. Aguiar I. Cardoso I. Correia Z. Correia T. (2021). Case report: Parental loss and childhood grief during COVID-19 pandemic. Frontiers in Psychiatry, 12, 626940. 10.3389/fpsyt.2021.626940 PMC7928371 33679484

[bibr30-10541373211038084] Scheinfeld E. Gangi K. Nelson E. C. Sinardi C. C. (2021). Please scream inside your heart: Compounded loss and coping during the COVID-19 pandemic. Health Communication, 1–13. 10.1080/10410236.2021.1886413 33586557

[bibr31-10541373211038084] Sher L. (2020). COVID-19, anxiety, sleep disturbance and suicide. Sleep Medicine, 70(January), 124. 10.1016/j.sleep.2020.04.019 32408252 PMC7195057

[bibr32-10541373211038084] Shigemura J. Morganstein J. C. Kurosawa M. Benedek D. M. (2020). Public responses to the novel in Japan : Mental health consequences and target populations. Psychiatry and Clinical Neurosciences, 74(277-283), 2019–2020. 10.1111/pcn.12988 PMC7168047 32034840

[bibr33-10541373211038084] Singer J. Spiegel J. A. Papa A. (2020). Preloss grief in family members of COVID-19 patients: Recommendations for clinicians and researchers. Psychological Trauma-Theory Research Practice and Policy, 12(S1), S90–S93. 10.1037/tra0000876 32478543

[bibr34-10541373211038084] Sowden R. Borgstrom E. Selman L. E. (2021). It's like being in a war with an invisible enemy”: A document analysis of bereavement due to COVID-19 in UK newspapers. PLoS One, 16(3), e0247904. 10.1371/journal.pone.0247904 PMC7932501 33661955

[bibr35-10541373211038084] Stroebe M. Schut H. (2010). The dual process model of coping with bereavement : A decade on. OMEGA-Journal of Death and Dying, 61(4), 273–289. 10.2190/OM.61.4.b 21058610

[bibr36-10541373211038084] Stroebe M. Schut H. (2021). Bereavement in times of COVID-19: A review and theoretical framework. OMEGA-Journal of Death and Dying, 82(3), 500–522. 10.1177/0030222820966928 PMC7756059 33086903

[bibr37-10541373211038084] Stroebe M. Schut H. Stroebe W. (2007). Health outcomes of bereavement. Psychology and Health, 370 (9603), 1960–1973. https://doi.org/10.1016/S0140-6736(07)61816-9 10.1016/S0140-6736(07)61816-9 18068517

[bibr38-10541373211038084] Tang S. Q. Xiang Z. D. (2021). Who suffered most after deaths due to COVID-19? Prevalence and correlates of prolonged grief disorder in COVID-19 related bereaved adults. Globalization and Health, 17(1), 1–9. 10.1186/s12992-021-00669-5 PMC7877329 33573673

[bibr39-10541373211038084] Vázquez Bandín C. (2020). Only the living can witness the passing of death: Mourning in times of pandemic. The Humanistic Psychologist, 48(4), 357–362. 10.1037/hum0000225

[bibr40-10541373211038084] Verdery A. M. Smith-Greenaway E. Margolis R. Daw J. (2020). Tracking the reach of COVID-19 kin loss with a bereavement multiplier applied to the United States. Proceedings of the National Academy of Sciences of the United States of America, 117(30), 17695–17701. 10.1073/pnas.2007476117 32651279 PMC7395491

[bibr41-10541373211038084] Vindegaard N. Benros M. E. (2020). COVID-19 pandemic and mental health consequences: Systematic review of the current evidence. Brain Behavior and Immunity, 89(May), 531–542. 10.1016/j.bbi.2020.05.048 32485289 PMC7260522

[bibr42-10541373211038084] Wallace C. L. Wladkowski S. P. Gibson A. White P. (2020). Grief during the COVID-19 pandemic: Considerations for palliative care providers. Journal of Pain and Symptom Management, 60(1), e70–e76. 10.1016/j.jpainsymman.2020.04.012 PMC7153515 32298748

[bibr43-10541373211038084] Walsh F. (2020). Loss and resilience in the time of COVID-19: Meaning making, hope, and transcendence. Family Process, 59(3), 898–911. 10.1111/famp.12588 32678915 PMC7405214

[bibr44-10541373211038084] Weinstock L. Dunda D. Harrington H. Nelson H. (2021). It's complicated-adolescent grief in the time of COVID-19. Frontiers in Psychiatry, 12, 638940. 10.3389/fpsyt.2021.638940 PMC7940762 33708148

[bibr45-10541373211038084] WHO. (2020). WHO Director-General's opening remarks at the media briefing on COVID-19—11 March 2020. Retrieved July 20, 2021, from https://www.who.int/director-general/speeches/detail/who-director-general-s-opening-remarks-at-the-media-briefing-on-covid-19–11-march-2020.

[bibr46-10541373211038084] Worldometer. (2021). COVID 19 Coronavirus Pandemic. Retrieved July 20, 2021, from: https://www.worldometers.info/coronavirus/

